# Quality of Care is Perceived to be High with Community-based Antiretroviral Therapy (ART) Services for Female Sex Workers in Tanzania: Qualitative Findings from a Pilot Implementation Science Study

**DOI:** 10.1007/s10461-023-04155-4

**Published:** 2023-08-26

**Authors:** Waimar Tun, Donaldson F. Conserve, Catherine Bunga, Kidola Jeremiah, Louis Apicella, Lung Vu

**Affiliations:** 1 Population Council, Social and Behavioral Sciences Research, 4301 Connecticut Ave., NW, Suite 280, Washington, DC 20008 USA; 2 Milken Institute School of Public Health, George Washington University, Washington, DC USA; 3 Mwanza Research Centre, National Institute for Medical Research, Mwanza, Tanzania; 4 The World Bank, Washington, DC USA

**Keywords:** Female sex workers, Community-based ART, Antiretroviral therapy, Quality of care

## Abstract

This qualitative study reports on female sex workers’ (FSWs) perceptions of the quality of antiretroviral therapy (ART) services they received as part of a community-based ART distribution intervention compared to services received by FSWs in the standard of care (SOC) arm. In-depth interviews were conducted with 24 participants to explore their perceptions of the quality of ART services. Data was analyzed using a quality-of-care framework that included but was not limited to, domains of accessibility, effective organization of care, package of services, and patient-centered care. Overall, FSWs in the intervention arm reported community-based ART services to be highly accessible, organized, and effective, and they highly valued the patient-centered care and high level of privacy. Community-based ART programs for FSWs can have high quality-of-care, which can have a positive effect on HIV treatment outcomes for FSWs.

## Introduction

Globally, female sex workers (FSWs) have lower levels of antiretroviral therapy (ART) uptake and poor treatment outcomes [1, 2]. In Tanzania, a study among community-recruited FSWs indicated that only 31% were previously aware of their HIV status, out of which 69% were on ART. Of those on ART, 70% were virally suppressed [3]. There are many challenges to ensuring access to and quality of ART services. These include lack of time, lack of privacy, cost to patients attending ART clinics (e.g., transportation, food, lost income), and poor provider attitudes and behaviors [4–6]. This is particularly salient for FSWs since they face multiple layers of stigma and discrimination related to their behaviors and lifestyles when accessing health services [7, 8].

There is growing evidence that community-based HIV treatment services can be an effective strategy for ensuring FSWs living with HIV initiate and are retained in ART services, and for reducing the burden for both patients and facility staff [9–15]. Community-based models vary and may include the use of outreach, mobile services, community drug distribution points, drop-in centers, peer navigation, home-based ART, and community ART groups (with a client-led drug pick-up rotation system). Recent reviews by Atuhaire et al. and Ibiloye et al. [9, 15] on community-based interventions for key populations across the HIV care cascade in sub-Saharan Africa found that there was improved ART use and retention, although the effect varied depending on the package of interventions. For example, ART use was improved when services were provided in facilities on a daily basis in hotspots with flexible hours or when interventions included adherence support and text message follow-ups. Additionally, Atuhaire et al. found that ART use could not be sustained over longer follow-up periods [9].

With the increasing evidence of improved ART initiation and retention among FSWs served by community-based ART services in sub-Saharan Africa, there is a need to also understand FSWs’ perceptions of the quality of services provided through community-based ART delivery programs. It is important to understand whether quality of services as perceived by patients may be compromised if services are provided outside of facility-based care. Such evidence was particularly needed in Tanzania at the time of the study as the country was commencing a more community-based ART service-delivery approach utilizing community health workers [16].

This qualitative study reports on FSWs’ perceptions of the quality of ART services they received as part of a community-based ART distribution implementation science study compared to FSWs in the comparison group [17]. Project SOAR (led by the Population Council) and the *Sauti* (“Voices”) project (led by Jhpiego) designed an intervention and evaluation to assess the increase in initiation and retention in ART when using community-based HIV testing and counseling plus (CBHTC+) platforms (mobile and home-based) [18].

## Methods

### Study Design

This qualitative study was conducted as part of the overall evaluation of a pilot implementation science study to provide ART services via a community-based platform [17, 19]. The evaluation of the pilot study entailed a quasi-experimental prospective cohort study whereby participants in the intervention and comparison arms were followed-up at 6 and 12 months. Semi-structured in-depth interviews (IDIs) were conducted with FSWs in the community-based ART intervention and a comparison arm after 12 months of the intervention implementation to compare FSWs’ perceptions of the quality of ART services.

### Theoretical Framework for the Study

The study was guided by a quality-of-care framework developed by Becker and colleagues [20]. The framework was informed by the Bruce-Jain quality-of-care framework [21] and the Sofaer and Firminger quality of health services framework [22], and consisted of eight domains (accessibility, client-staff interactions, effective organization of care, perceived technical quality and competency, communication and information, structure and facilities, package of services, and patient-centeredness) detailed in Table 1.

**Table 1:** Eight domains of quality of care

Domains	Definition	
Accessibility	Services are geographically accessible, affordable, and convenient in place and time.	
Client-staff interactions	Providers and staff demonstrate respect, courtesy, friendliness and empathy, and respect clients’ privacy.	
Effective organization of care	Care is organized in terms of enrollment/registration, waiting time, follow-up, timely test results, and referrals; care is coordinated among providers/facilities.	
Perceived technical quality and competency	Perception by clients that providers are knowledgeable, competent, and experienced.	
Communication and information	Information provided to clients is understandable and sufficient; clear explanation of complex technical information; provision of information on status, prognosis, processes of care; timely test results	
Structure and facilities	Environment where care is provided is comfortable, safe, clean, and private/secure.	
Package of services	Clients receive appropriate bundle of services, including counseling and referrals, that fulfill their needs. (In the case of ART services, are clients provided HIV-related services such as HIV prevention, and services related to TB, STIs, and family planning?)	
Patient-centeredness	Clients receive individualized care that is tailored to the needs and preferences of clients.	

### Study site

The study was conducted in Njombe (intervention) and Mbeya (comparison) regions in Tanzania. These two regions are part of the Sauti project—a large USAID-funded HIV-prevention program for key populations led by Jhpiego, the implementing partner of this research project. Njombe was purposely selected because of its high HIV burden—32.9% among FSWs in Iringa, which Njombe was part of until 2012 [23], a high estimated number of FSWs (3,871) [24], and its designation as a priority region by the donor. Mbeya region was selected as the comparison site as Sauti was similarly operating its community-based HIV testing and counseling (CBHTC+) mobile and home-based platforms in the region and it had a comparably high HIV prevalence of 29.2% among FSWs [23], and a high number of FSWs (10,152) [24].

### Description of the Intervention

The community-based ART intervention was implemented in Njombe region. ART services were provided in a place convenient for FSWs (usually at home) by a team consisting of a clinician, nurse, and peer counselor. Those in the comparison arm were referred to government ART Care and Treatment Centres (CTCs) following national guidelines. The study found that community-based ART distribution can lead to higher ART initiation with continued ART use and better adherence after six months, compared to facility-based ART provision [17]. Details of the ART services are outlined in Table 2.

**Table 2:** Services provided in the intervention and comparison arms

Intervention Arm (Njombe)	Comparison Arm (Mbeya)
Testing When: Daily/moonlight, walk-ins Where: Community locations—mobile tents, rented rooms, or FSWs’ home. Service provided close to community; easy to access, less crowded, flexible time/days. Provider: Nurses and clinicians, peer educator mobilization	Testing When: Daily/moonlight, walk-ins Where: Community locations—mobile tents, rented rooms, or FSWs’ home. Service provided at limited number of static CTC facilities. Longer commuting, and often more crowded, fixed working hours/days. Provider: Nurses and clinicians, peer educator mobilization
Linkage to ART Same day enrollment in care through CBHTC + sites Standard three sessions of adherence counseling (1 day) Provider: Nurses and doctor	Linkage to ART Referral given to government CTC facilities same day as HIV testing Standard three sessions of adherence counseling (3 consecutive days) Provider: Counselor and doctor
**ARV supply and refill** One-month supply at first visit; two-month supply at Month 1 visit; 3-month supply at third visit and thereafter Where: Community locations—mobile tents, rented rooms, homes Provider: Nurse and doctor	**ARV supply and refill** Typically, one-month supply. If deemed stable by doctor after first six months, a patient may be given drug refill appointments at 2–3 month intervals. Where: Government facilities Provider: Doctor
**Average number of clients seen per day for ART services**: 10–15 clients for both nurses and clinician **Average time per client**: 15 min by clinician and 15–60 min by nurse	**Average number of clients seen per day for ART services**: 30–50 clients for nurses and clinicians **Average time per client**: 10–20 min by clinician and 5–30 min by nurse
**Follow-up**: Peer educators stayed in regular contact with participants through text messaging and WhatsApp messaging and met them monthly to assess their adherence, side effects, and provide peer support.	**Follow-up**: No follow-up contact in between CTC visits
**Services Provided in Both Arms**	
Standard client assessment and referrals to family planning and other services (TB screening and testing, STI testing and treatment, mental health support; and gender-based violence services)	
Viral load and CD4 testing according to national guidelines	
Enhanced adherence counseling (3 sessions) when treatment failure is suspected; this is conducted by a counselor to help clients overcome factors contributing to treatment failure	

### Study Participants and data Collection Procedure

A cohort of 617 FSWs living with HIV not currently on ART (309 in the intervention arm and 308 in the comparison arm) was recruited between July and October 2017 through brochures, announcements during outreach activities, announcements at targeted health facilities in the catchment area, drop-in centers, CBHTC + sites, peer support meetings, and HIV counseling sessions. Details of the main study are reported elsewhere [17, 19]. Briefly, eligible participants for the main study were people living with HIV as identified through community-based testing sites, were females aged 18 years or older who reported a history of selling sex for money in the past 6 months, were not on ART, were planning to stay within the catchment area for the next 12 months, and were willing to provide informed consent. Participants in the intervention arm were registered into HIV care through the CBHTC + services right away if they chose to initiate ART. Participants in the comparison arm were given a referral card with a list of available government ART facilities; clients could be escorted to services if they desired. Participants completed a baseline and follow-up (6 and 12 months) interviewer-administered structured questionnaire.

Semi-structured IDIs were conducted in Swahili with a subsample (12 from each arm) of the cohort retained in the study at 12 months to compare quality of care from the perspectives of the ART clients. The 12-month retention was 85.8% in the intervention arm and 85.1% in the comparison arm. We stratified participants by ART status (current ART usage or not) and selected a random sample. When a participant was not reachable, a replacement was selected. Although we tried to ensure representation of clients currently on ART as well as those not on ART, most participants ended up being those who were on treatment since the majority of participants were currently on ART at 12 months, particularly in the intervention arm. The interviewees were recorded and asked a series of open-ended questions that explored their perspectives on the eight domains of quality of care of ART services.

### Data Analysis

Semi structured interviews were transcribed and translated into English. Transcripts were then analyzed using the quality-of-care framework (accessibility, client-staff interactions, effective organization of care, perceived technical quality and competency, communication and information, structure and facilities, package of services, patient-centeredness) [20]. A directed content analysis—using a deductive lens—was used by sorting the data into the general topical domains of the quality-of-care framework [25, 26]. Team members discussed the domains of the quality-of-care framework to come to a common understanding of the different domains. The team then reviewed the contents to analyze FSWs’ perspectives on the quality-of-care domains. Regular team meetings were organized to discuss and resolve disagreement in categorization of the text. The perspectives of participants regarding the quality of services they received were summarized for each of the domains of the quality of health services framework where they appear.

#### Ethical Considerations

##### Ethical Approval

was obtained from Tanzania’s Medical Research Coordinating Committee (MRCC), National Institute for Medical Research, the Mbeya Zonal Consultant Hospital, Mbeya Medical Research and Ethics Review Committee (Tanzania), and the Population Council Institutional Review Board (United States). Written informed consent was obtained from all participants in Swahili before conducting any study-related activities.

## Results

Participants in the intervention group (median: 29 years) were slightly younger than those in the comparison group (median: 33 years), and there was a higher number of single/unmarried participants in the intervention group compared to the comparison group (Table 3). While all 12 participants in the intervention arm were currently on ART, 9 out of 12 in the comparison arm were on ART. Lastly, only one reported discontinuing ART for more than 30 days in the last 6 months in the intervention.

**Table 3:** Characteristics of IDI participants in the intervention and comparison arms

Characteristics	Intervention n (N = 12)	Comparison n (N = 12)
Median age (Interquartile range)	29 (25, 31)	33 (29,40)
Marital status Single/Unmarried Married Divorced Widowed	10 0 1 1	3 0 4 5
Current ART use	12	9
Discontinued ART for more than 30 days in last 6 months	1	3

Findings are organized by the eight domains of quality of service.

### Community-based ART participants reported easy access, while comparison arm participants reported challenges in access to ART services

Many participants in the intervention reported on the *convenience* of having antiretroviral medications (ARVs) delivered to them at a location of their preference, which helped them not only to save *time* (i.e., travel time and waiting time at clinic), but also removed *transportation barriers* associated with the travel costs. Several spoke about their busy schedules with work and that the clinic visits with the ART providers didn’t take time away from their jobs. Those in the comparison arm had to travel to the ART facilities and wait for services there. The following quote describes the convenience participants experienced through the intervention:

*It is a quick service; one can carry on [with] their jobs while still receiving treatment. And you can get treated when contacting them at any time in case you get sick or have an emergency. They can follow you at a convenient location for treatment, so I don’t have to attend clinic.* (Intervention, Age 36)

Another community-based ART participant described how she experienced challenges with paying for transportation to the clinic prior to the community-based ART intervention:

*I was happy with home service. It’s usually hard for us to find fare and come for medication. We live far in the villages.* (Intervention, Age 35)

Indeed, participants in the comparison group reported challenges associated with accessibility related to lack of money to pay for transportation to the clinic or being afraid of people:

*“There are two things involved. Sometimes it’s about lack of money or being scared of people, like I told you before.“* (Comparison, Age 29)

One other comparison group participant shared how she sometimes had to rely on her friend to assist her with transportation in order to attend the clinic and collect her refill:

*The reason is lack of fare. I sometimes have no option. But sometimes my friend would give me money to go get the pills.* (Comparison, Age 29)

A few in the comparison group indicated that it was easier for them to access their ARVs once they shifted to a 2-month refill schedule. It became less of a burden with regard to time and cost.

*I was very happy with the arrangement of providing drugs for two months; it saves a lot of time.* (Comparison, Age 29)

### Participants in both arms reported satisfaction with client-staff interactions

Participants from both arms expressed similar sentiments regarding client-staff interactions. Both groups reported that interactions with their respective providers were courteous, respectful, helpful, supportive, and non-stigmatizing. One participant from the intervention stated:

*The service providers don’t use offensive language around us. They are confidential, and while they bring us drugs they also guide on the proper use of ARVs.* (Intervention, Age 26)

Similarly, a participant from the comparison group described the wonderful interaction she had with the providers at the clinic:

*Without telling lies, we have wonderful conversations with them.... There was a time I was a bit sick and went there to report; service providers served me well.* (Comparison, Age 40)

### The organization of care in the community-based ART services was deemed to be highly efficient

Participants in the intervention arm spoke highly about the short wait time required to see providers and the efficiency in receiving viral-load test results. However, participants spoke poorly about these issues in the government clinics.

#### Wait time to see providers

Intervention participants spoke highly of the fact that they saved a great deal of time from not having to wait at the government clinics to receive services. Those in the intervention arm indicated that they were able to get services at a time that was convenient to them and that they did not have to waste time traveling to and waiting in the government clinic. FSWs in the comparison group, on the other hand, encountered long waits to be seen by the providers.

*Some days can be filled with lots of people [at government clinic] so we’ll be forced to wait and stay in queue till 2pm.* (Comparison, Age 29)

*So it really helps a lot. I may just tell them to bring me drugs at my preferred rendezvous point.* (Intervention, Age 36)

One participant mentioned that the enrollment process at the government clinics took so long that it deterred her from registering for care.

*“I’ve once been in the hospital before joining this [community ART] program. I wanted to start on ARVs, but they had such a long enrollment procedure…so I changed my mind.”* (Intervention, Age 30)

#### Viral-load testing

Participants in the community-based ART intervention spoke about receiving their viral-load test results in a timely manner, allowing them to know how they were responding to the treatment. However, most of the participants in the comparison group indicated that although they did have their viral-load test conducted, they never learned the result.

*I understand [what viral load is] because I also got tested and they got my results back. I’m thankful that they check my progress to see if I have either low or high viral load. They tell me I have good progress each time they test me, so I’m thankful for that.* (Intervention, Age 36)

*I like how they give my results back after taking blood samples. Leads me to know the progress of my health, if I have a high or low viral count.* (Intervention, Age 25)

*Since I tested, though, I have never gotten the results back; they have not even written results in the card*. (Comparison, Age 40)

### Technical quality and provider competency were perceived to be high in both arms

Participants from both the intervention and comparison groups reported that they perceived the care they received to be technically competent and the providers to be competent, experienced, and knowledgeable about how to treat them for HIV.

*Yes, they are knowledgeable. I see them as professional nurses who know what they do. I don’t see any difference from nurses in the hospitals. They test the same way, and services are similar to those in hospitals.* (Intervention, Age 36)

Referring to the providers, one participant from the comparison group stated, “*Yes, they are knowledgeable.”* Two other participants from the comparison group continued describing how the providers educated them about how to use ARVs and live a healthy lifestyle.

*Mostly they teach us the proper use of drugs, eating a balanced diet, such as vegetables and oils, nuts, and milk once in a while, etc. Most important is the time for taking drugs.* (Comparison, Age 36)

*They were educating us during the first time, so we got used to it; we just attend there to queue in line and get provided with drugs and some other services, in case we are sick or something like that. They usually get to ask us if we have any health-related problems.* (Comparison, Age 29)

### Participants in both arms were satisfied with the communication and information they received

Participants in both arms spoke highly of the quality, comprehensiveness, and usefulness of the information that was communicated to them by their respective service providers. They reported receiving updates about their health, disclosure to partner, healthy lifestyle including ways not to infect others or get reinfected, the meaning of viral-load testing, returning for follow-up appointments, and most important, how to adhere to the ARV medications.

*They helped me rightly First of all, they advised me on doing the right things in order to avoid any further infections, as well as avoid infecting other people.* (Intervention, Age 36)

*They also advised me against unprotected sex, to refrain from stress and deep thoughts I should eat a balanced diet, and I should take [an antibiotic] for other infectious diseases.* (Intervention, Age 26)

*The service is good because they explain and teach many things. For example, the young man who usually serves me, he tells me about my medications and the series of things I could encounter along the way.* (Comparison, Age 40)

Participants in both arms spoke about the emphasis the providers placed on the issue of adherence to the ARV medications. Many in both arms spoke about the repeated messages around adherence they received from the providers. They understood the importance of taking ARVs daily and at the same time.

*We are being told that using ARVs deactivates HIV and activates the virus when we stop taking the meds, causing the virus to multiply as well. That’s why we are advised to adhere to drugs on time. If it’s at 8pm, or 9pm, it should be constant. (Comparison, Age 36)*

Participants in both arms reported that they rarely missed their dose and knew the importance of adherence. Almost all participants reported that they were able to take their ARV medications regularly and rarely forgot to take them.

*I have never forgotten my dear, I feel like that’s my life; even my kids have noticed how well I’m doing, and I thank God for this good progress.* (Comparison, Age 40)

*I believe taking these ARVs is my life, and I’ll die if I stop. So that kind of thinking gives me strength to take them every day as if it’s my life, my food.* (Intervention, Age 30)

On the occasions that they did not take their medications on time or missed their doses, it was often due to fears about others learning of their HIV status or their lifestyle that made it challenging to take their ARVs regularly (e.g., spending the night elsewhere or traveling).

### Intervention participants appreciated the privacy afforded by the community-based ART services

The concept that was discussed the most with regard to the structure and facilities of ART services was that of privacy. Intervention participants valued the privacy they were able to have through community-based ART services.

*There is privacy, and no long queues. It’s just them and me, and I liked that arrangement [referring to the community-based ART intervention].* (Intervention, Age 36)

Several participants described not wanting to receive ART services from government facilities due to a lack of privacy. They specifically feared running into people who might know them and the HIV-related stigma they would face from community members when attending services at the government facilities.

*A lot of people may know you at the hospital [in reference to receiving care at hospitals].* (Intervention, Age 30)

*With the services, I’d really like if they were able to deliver the drugs to us, because a lot of people are scared to attend health centers openly in fear that other people will know about their HIV status and spread the news in the street. A lot of people lose hope and die without medication. People would soon know that you have HIV/AIDS as long as you go to that section of the health center.* (Comparison, Age 40)

### Participants in both arms were satisfied with the package of services offered

Part of providing quality services entails providing not only ART services but also a range of other services that are important for people living with HIV and engaging in higher-risk sexual behaviors. Most participants in both arms reported that they received appropriate services and that providers discussed and provided a range of services that were particularly important for clients involved in sex work, including HIV prevention, family planning, sexually transmitted infections (STIs), and cervical cancer screening.

*They also tested me on cervical cancer and results were negative.... They just gave me condoms and advised that I should be using them to avoid new infections*. (Intervention, Age 30)

*They told me about it [other health issues], and I got tested for STDs and TB. Results were negative for both. They told me to use protection when having sex. They insist on disengaging [from drinking alcohol], unprotected sex, and other things.* (Comparison, Age 28)

*They said I could join contraception programs using injections, pills, hormonal rings, or an intrauterine device.* (Intervention, Age 30)

*They told me to use protection against STIs, since I would never know if people I’m having sex with have infections or not, and hence I’d be subjected to acquiring newer infections. So they advised me to get tested frequently, and I agreed to that request.* (Intervention, Age 36)

### Community-based ART clients praised the patient-centered care they received

There was a marked difference between intervention and comparison participants in the way they spoke about the individualized and tailored care they received from their respective ART care teams. Many intervention participants spoke about the frequent follow-ups made by the community-based ART team to check up on how they were doing and the amount of ARVs remaining, remind them of upcoming visits, and set up follow-up visit appointments. Intervention participants particularly appreciated how the community-based ART team would accommodate their busy schedules by being willing to meet at a convenient time and place. This made them feel like the providers listened and cared about their individual well-being. On the other hand, while those in the comparison group expressed that they were satisfied with the services from their providers, none of the participants spoke about receiving this type of individualized care.

*I liked it because they [community-based ART team] are close to the clients, and they do follow-ups to monitor clients’ progress. Since it really helps a lot, I may just tell them to bring me drugs to my preferred rendezvous point. This service is very good compared to the alternative of not knowing what to do. They monitor progress and take tests frequently upon use of medication, so I think it’s the best.* (Intervention, Age 36)

*Among others, is that they usually call to check on my progress, and the amount of ARVs that remain. I’m really happy with how they care about me.* (Intervention, Age 29)

## Discussion

This qualitative evaluation of the quality of ART services found that FSWs in the community-based ART arm highly valued the accessibility, privacy, effective organization of care, and patient-centered care they received through the community-based ART delivery model, while those receiving services through the government facilities spoke less highly of these features. However, FSWs in both arms were equally satisfied with the quality of client-staff interactions, communication and information, technical quality, and package of services provided. This is promising for community-based ART programs and indicates that these programs can offer high-quality services. This is particularly pertinent in Tanzania as they incorporate community-based HIV testing and treatment services as part of the national program.

The findings reported here support the quantitative results of the evaluation of this community-based ART pilot which revealed better treatment initiation and 12-month retention (self-reported current use of ART) in the intervention arm compared to the comparison arm [27]. Specifically, the quantitative findings showed that those in the community-based ART intervention were more likely to initiate ART (AOR: 19.0; 95% CI: 4.4–81.6) and be retained in ART services after 12 months (AOR: 16.0; 95% CI: 4.6–55.7). The findings from this qualitative analysis shed light on why initiation may be better with community-based ART services. In particular, participants pointed to the long wait and registration process to initiate ART at CTC facilities. With the community-based ART intervention, participants received immediate adherence counseling and a one-month ARV supply, whereas those in the comparison arm had to not only wait to be seen but also undergo a lengthy enrollment process. Our findings also explain the greater retention in the intervention group; those in the intervention spoke about the patient-centered care they received with active reminders and follow-up. This likely allowed them to receive their ARVs on time without any treatment gaps. Providing patient-centered care was likely easier in the intervention arm given that this was implemented in the context of a study and the community-based ART team attended to far fewer clients per day compared to providers at the CTC facilities. The burden of finding the time to get to and attend appointments is shifted to the community-based ART providers (as opposed to the clients); providers prioritized follow-up calls and even waited for clients if clients were late to appointments. It is possible that when conducted at scale with more clients, community-based ART providers may have less ability to manage time, which was an important component of providing this high level patient-centered care. However, various strategies may be used to maintain high levels of patient-centered care, such as employing lay workers to conduct follow-up calls and using community-based ART distribution points to increase efficient use of provider time, and other task-shifting activities [28].

Last, despite better retention in the intervention arm, the quantitative findings from the pilot did indicate that viral suppression among those retained in ART was similar between the two arms (80–85% at 6- and 12-month follow-up) [27]. Thus, once on ART, clients were able to adhere to ART and attain viral suppression, regardless of service-delivery model. Our qualitative findings here support this finding and show that participants in both arms were receiving strong messages regarding adherence. In fact, both participant groups reported strong motivation to adhere. The one difference regarding viral-load testing between the two groups was that those in the community-based intervention were more likely to have received viral-load testing and results. Thus, there is a need to improve on reporting viral-load test results back to clients in CTC facilities. If feasible, point-of-care viral-load testing may be the solution to ensuring results can be provided the same day as the follow-up visit given that individualized follow-up is difficult with CTC services.

The quality of staff-client interaction in the two arms was found to be similar. Participants in both arms reported being treated with respect by providers. This is not a surprising finding given that the government has prioritized in its National HIV and AIDS Strategic Plan (2017–2022) the training of health-care workers throughout the country on the provision of key and vulnerable population (KVP)-friendly services using the national training curriculum for KVP-friendly services [29]. The curriculum includes sensitization of health-care workers on how to provide services to key populations, including FSWs, without stigma and judgmental attitudes. The curriculum also includes training providers on the comprehensive package of clinical and counseling services needed particularly for high-risk populations. This also explains why the package of services received by FSWs in both arms was similar. Both intervention and comparison arms reported receiving counseling and care for issues that are particularly critical for FSWs (e.g., STIs, cervical cancer screening, and family planning). However, cervical cancer screening was mentioned by only one person out of the 24 participants. Training for health-care workers will need to emphasize cervical cancer screening more, especially for those who serve higher-risk populations.

Although CTC clients reported receiving friendly and non-stigmatizing services from providers, anticipated HIV-related stigma (e.g., FSW’s fears of community members at health facilities learning their HIV status) remains a barrier to accessing ART services through CTC. Participants in the comparison group clearly expressed their hesitancy in accessing ART through CTC for fear of encountering people who might recognize them. The particular concern was that others would learn that they were living with HIV as opposed to their engagement in sex work. While anticipated HIV-related stigma is a concern even for non-sex workers, it may be more of a concern for sex workers as they may fear that their clients would learn of their HIV status when receiving services at health facilities. Intervention participants, on the other hand, pointed to the benefits of receiving services through the community-based program as it afforded them greater privacy. Thus, a key benefit of community-based ART services appears to be the ability to overcome a major obstacle in ART access—that of anticipated HIV-related stigma. Interestingly, stigma related to sex work when receiving services at the government CTC facilities was never mentioned by any participants. Again, this is likely due to the key population sensitization training received by CTC staff.

While many studies have reported on HIV-treatment-related outcomes from community-based ART services for FSWs, only one study has reported on FSW’s perspectives on quality of services of the community-based service delivery model for FSWs in sub-Saharan Africa. The findings from this study contrast with the findings from a study reporting on FSW’s perspectives on differentiated services delivery models in Uganda [10]. FSWs in the Uganda study preferred the facility-based service-delivery model over the community-based models, specifically the community client-led ART delivery model (CCLAD) and community drug distribution points (CDDP) at drop-in centers. FSWs in the Uganda study did not prefer the community-based model because of stigmatization and discrimination, lack of privacy and confidentiality, and the limited package of services offered. The community-based models in the Uganda study differed greatly from this study. For example, in the CCLAD model, ART clients were part of a group with other ART users, which may explain concerns about lack of confidentiality. Additionally, the Uganda models were less private since services were provided at drop-in centers or open spaces in the community. This is unlike the points of service in this study, which were often in a mobile tent or a participant’s home. Another reason for the difference between these two studies is the negative health worker’s attitude of the community-based services in Uganda. One main similarity between these two studies was the high level of accessibility the community-based models provided—convenient locations close to where they work or stay, no need for transport money, flexible times, and availability of service staff. The similarities and differences between these two studies highlight the need to tailor community-based services to ensure that they meet the specific needs of the target population.

While this study found a high quality of services in the community-based ART model, it is important to acknowledge that the perceived quality of the community-based ART services is not necessarily only beneficial to the FSW population; it is beneficial to all living with HIV and wanting ART services. However, implementation of such a program would be more cost-effective if the intervention was targeted at those at highest risk of HIV transmission, including FSWs. The community-based model may need to be tailored for different populations to ensure acceptability and effectiveness.

This study has several limitations. First, the views of providers are not included, hence their perspectives on the feasibility of the intervention are not accounted for here. However, although their perspectives are missing, we have a detailed comparison of services provided in the two arms, which gives insight into some implementation issues (e.g., workload, patients seen per day). Second, due to the high retention rate in the intervention arm, we were not able to enroll participants who were lost to follow-up from treatment. Last, the small sample size of the study with only 12 in each arm may limit the richness of the qualitative data, limiting the variations observed.

## Conclusions

In the past decade, Tanzania has recognized the importance of community-based HIV services, including for care and treatment. In fact, community-based services have been included as an integral part of the management of HIV and AIDS in the latest national guidelines [16]. The government continues to emphasize the importance of strong collaboration between community-based programs and health facilities to ensure a continuum of comprehensive and integrated services for clients on treatment. The findings from this study highlight the high service quality in the community-based ART service-delivery model. In particular, the community-based ART model is perceived to be of high quality on a few critical features that improve ART initiation and retention—namely that of accessibility, effective organization of care (i.e., time saved, convenience, and viral-load testing results), service environment to overcome HIV-related stigma, and patient-centered care that is particularly conducive to greater retention. Thus, these findings, along with the findings from the pilot evaluation of increased ART initiation and retention, indicate that the community-based intervention may best be provided for FSWs and other populations that face challenges with ART initiation and are at high risk of loss to follow-up.

## Data Availability

Full transcripts are not made publicly available given the sensitivities to protect the confidentiality of respondents from this stigmatized population group. Interview data that supports the findings from this analysis can be made available upon reasonable request to the Principal Investigator Waimar Tun at wtun@popcouncil.org.

## Code Availability

Codes are available upon reasonable request to the Principal Investigator Waimar Tun at wtun@popcouncil.org.

## References

[1] Mountain E, Mishra S, Vickerman P, Pickles M, Gilks C, Boily MC (2014). Antiretroviral therapy uptake, attrition, adherence and outcomes among HIV-infected female sex workers: a systematic review and meta-analysis. PLoS ONE.

[2] Mountain E, Pickles M, Mishra S, Vickerman P, Alary M, Boily MC (2014). The HIV care cascade and antiretroviral therapy in female sex workers: implications for HIV prevention. Expert Rev Anti Infect Ther.

[3] Kerrigan D, Mbwambo J, Likindikoki S, Beckham S, Mwampashi A, Shembilu C (2017). Project Shikamana: baseline findings from a community empowerment-based combination HIV Prevention Trial among Female Sex Workers in Iringa, Tanzania. J Acquir Immune Defic Syndr.

[4] Tuller DM, Bangsberg DR, Senkungu J, Ware NC, Emenyonu N, Weiser SD (2010). Transportation costs impede sustained adherence and access to HAART in a clinic population in southwestern Uganda: a qualitative study. AIDS Behav.

[5] Duff P, Kipp W, Wild TC, Rubaale T, Okech-Ojony J (2010). Barriers to accessing highly active antiretroviral therapy by HIV-positive women attending an antenatal clinic in a regional hospital in western Uganda. J Int AIDS Soc.

[6] Mshana GH, Wamoyi J, Busza J, Zaba B, Changalucha J, Kaluvya S (2006). Barriers to accessing antiretroviral therapy in Kisesa, Tanzania: a qualitative study of early rural referrals to the national program. AIDS Patient Care & STDs.

[7] Scorgie F, Nakato D, Akoth DO, Netshivhambe M, Chakuvinga P, Nkomo P (2011). I expect to be abused and I have fear”: sex workers’ experiences of human rights violations and barriers to accessing healthcare in four african countries.

[8] Nakanwagi S, Matovu JK, Kintu BN, Kaharuza F, Wanyenze RK. Facilitators and barriers to linkage to hiv care among female sex workers receiving hiv testing services at a community-based organization in Periurban Uganda: A Qualitative Study. Journal of sexually transmitted diseases. 2016;2016. 10.1155/2016/7673014 PMC4963560 27493826

[9] Atuhaire L, Adetokunboh O, Shumba C, Nyasulu PS (2021). Effect of community-based interventions targeting female sex workers along the HIV care cascade in sub-saharan Africa: a systematic review and meta-analysis. Syst Rev.

[10] Atuhaire L, Shumba CS, Nyasulu PS (2022). My condition is my secret”: perspectives of HIV positive female sex workers on differentiated service delivery models in Kampala Uganda. BMC Health Serv Res.

[11] Bemelmans M, Baert S, Goemaere E, Wilkinson L, Vandendyck M, van Cutsem G (2014). Community-supported models of care for people on HIV treatment in sub-saharan Africa. Trop Med Int Health.

[12] Decroo T, Telfer B, Dores CD, White RA, Santos ND, Mkwamba A (2017). Effect of Community ART Groups on retention-in-care among patients on ART in Tete Province, Mozambique: a cohort study. BMJ Open.

[13] Kerrigan D, Mbwambo J, Likindikoki S, Davis W, Mantsios A, Beckham SW (2019). Project Shikamana: community empowerment-based combination HIV Prevention significantly Impacts HIV incidence and care continuum outcomes among female sex workers in Iringa, Tanzania. J Acquir Immune Defic Syndr.

[14] Leddy AM, Mantsios A, Davis W, Muraleetharan O, Shembilu C, Mwampashi A (2020). Essential elements of a community empowerment approach to HIV prevention among female sex workers engaged in project Shikamana in Iringa, Tanzania. Cult Health Sex.

[15] Ibiloye O, Masquillier C, Jwanle P, Van Belle S, van Olmen J, Lynen L (2022). Community-based ART service delivery for key populations in Sub-Saharan Africa: scoping review of outcomes along the Continuum of HIV Care. AIDS Behav.

[16] Ministry of Health (2017). Community Development, gender, Elderly, and children /National AIDS Control Programme (Tanzania). National Guidelines for the management of HIV and AIDS.

[17] Tun W, Apicella L, Casalini C, Bikaru D, Mbita G, Jeremiah K (2019). Community-based antiretroviral therapy (ART) delivery for female sex workers in Tanzania: 6-month ART initiation and adherence. AIDS Behav.

[18] Mbita G, Mwanamsangu A, Plotkin M, Casalini C, Shao A, Lija G (2020). Consistent condom use and dual protection among female sex workers: surveillance findings from a large-scale, community-based combination HIV prevention program in Tanzania. AIDS Behav.

[19] Vu L, Tun W, Apicella L, Casalini C, Makyao N, Tsang S (2020). Community-based antiretroviral therapy (ART) delivery for female sex workers in Tanzania: intervention model and baseline findings. AIDS Care.

[20] Becker D, Koenig MA, Mi Kim Y, Cardona K, Sonenstein FL (2007). The quality of family planning services in the United States: findings from a literature review. Perspect Sex Reprod Health.

[21] Bruce J (1990). Fundamental elements of the quality of care: a simple framework. Stud Fam Plann.

[22] Sofaer S, Firminger K (2005). Patient perceptions of the quality of health services. Annu Rev Public Health.

[23] Ministry of Health and Social Welfare, National AIDS Control Programme (Tanzania) (2014). HIV and STI Biological and behavioral survey, 2013 - a study of female sex workers in seven regions: Dar es Salaam, Iringa, Mbeya, Mwanza, Shinyanga, Tabora and Mara.

[24] Ministry of Health and Social Welfare, National AIDS Control Programme (Tanzania) (2014). Consensus estimates on Key Population size and HIV Prevalence in Tanzania.

[25] Hsieh H-F, Shannon SE (2005). Three approaches to qualitative content analysis. Qual Health Res.

[26] Saldaña J. The Coding Manual for qualitative researchers. SAGE Publications Ltd; 2021.

[27] Vu L, Tun W, Apicella L, Kidola J, Casalini C, Mbita G (2019). Community-based HIV treatment service delivery model for female sex workers in Tanzania: evaluation findings.

[28] UNAIDS, Medecins Sans Frontiers (2015). Community-based antiretroviral therapy delivery.

[29] National AIDSC, Programme (2017). Ministry of Health, Community Development, gender, Elderly and Children, Government of Tanzania. Health Sector HIV and AIDS Strategic Plan 2017–2022 (HSHSP IV).

